# A meta-analysis on the changes of BMI during an inpatient treatment with different follow-up lengths (short and long term) compared with the outpatient phase in obese patients

**DOI:** 10.1038/s41366-023-01297-2

**Published:** 2023-03-30

**Authors:** Mariangela Rondanelli, Clara Gasparri, Chiara Rigon, Cinzia Ferraris, Antonella Riva, Giovanna Petrangolini, Gabriella Peroni, Milena Anna Faliva, Maurizio Naso, Simone Perna

**Affiliations:** 1 IRCCS Mondino Foundation, Pavia, 27100 Italy; 2 Department of Public Health, Experimental and Forensic Medicine, University of Pavia, Pavia, 27100 Italy; 3 Endocrinology and Nutrition Unit, Azienda di Servizi alla Persona “Istituto Santa Margherita”, University of Pavia, Pavia, 27100 Italy; 4 Food Education and Sport Nutrition Laboratory, Department of Public Health, Experimental and Forensic Medicine, University of Pavia, 27100 Pavia, Italy; 5 Research and Development Unit, Indena, Milan, 20139 Italy; 6 Department of Biology, College of Science, University of Bahrain, Sakhir Campus P. O. Box, 32038 Zallaq, Kingdom of Bahrain

**Keywords:** Nutrition, Weight management

## Abstract

**Background:**

The management of obesity should be multidimensional based on the choice of the treatment and the intensity of the therapeutic-rehabilitative intervention. This meta-analysis aims to compare the changes on body weight and body mass index (BMI) during an inpatient treatment (hospitalized weight loss programs with different durations in terms of weeks) compared with the outpatient phase.

**Methods:**

The data obtained from the studies on inpatients have been layered into two categories: short term (studies with follow-up of max 6 months) and long term (studies with follow-up up to 24 months). Furthermore, this study evaluates which of the two approaches show the best impact on weight loss and BMI during 2 follow-ups at 6 to 24 months.

**Results:**

The analysis, which included seven studies (977 patients), revealed that the subjects underwent a short hospitalization had greater benefit, compared to those who were followed for a long time. The meta-analyzed mean differences for random effect (MD) showed a statistically significant decrease on BMI of −1.42 kg/m^2^ (95% CI: −2.48 to −0.35; *P* = 0.009) and on body weight −6.94 (95% CI: −10.71 to −3.17; *P* = 0.0003) for subjects who carry out a short hospitalization compared to outpatients. No reduction of body weight (*p* = 0.07) and BMI (*p* = 0.9) for subjects who undergo a long hospitalization compared to an outpatient.

**Conclusions:**

A short-term inpatients multidisciplinary weight loss program could be the best choice for the management of obesity and its related comorbidities; on the contrary, if the follow-up is of long duration, the significance is not confirmed. The hospitalization at the beginning of any obesity treatment is significantly better than only outpatients treatment.

## Introduction

Obesity has a negative impact on public health [1] because it is associated with long-term negative economic consequences [2].

Lifestyle modification is considered the standard of care and the first step in obesity management [3], followed by pharmacological therapy and bariatric surgery. Lifestyle changes are always desirable, already with a body mass index (BMI) between 25 and 30 kg/m^2^. Pharmacological therapy is indicated only in case of BMI > 30 kg/m^2^ or BMI > 27 kg/m^2^ in the presence of comorbidities [4]. Bariatric surgery is indicated only in case of BMI > 40 kg/m^2^ or BMI > 35 kg/m^2^ in presence of comorbidities [5].

In the management of obesity, the team approach is fundamental. It must be a multidimensional, interdisciplinary, multi-professional and integrated approach, involving medical doctors (internists, clinical nutritionists, psychiatrists, physiatrists), psychologists, dieticians, physiotherapists, and nurses [6].

Nutritional therapy can be set up in several ways, for example, as a low calorie (800–1200 kcal/day) very low-calorie diet (up to 800 kcal/day) or very low-calorie ketogenic diet (with a maximum 10% of carbohydrates, inducing ketosis).

The Mediterranean diet is an indispensable point of ref. [7]. Other types of dietary formulations, sometimes exasperated, such as hyperprotein, hypolipidic and hypoglucidic diets are to be considered with legitimate clinical scepticism as they are able to act on weight loss (but not specifically on body fat loss) in the short period of the beginning of the diet, but are of poor effectiveness and doubtful safety both in the short and long term [8].

The caloric restriction should be assessed based on the patient’s energy expenditure, preferably measured with indirect calorimetry, otherwise estimated with predictive formulas. It is recommended an energy restriction of between 500 and 1000 kcal compared to the daily energy expenditure calculated [8].

In the choice of the type of treatment, it is important to evaluate the setting and the intensity of the therapeutic-rehabilitative intervention [9], on the basis of medical comorbidity e psychiatric, disability and other factors perpetuation of the problem and risk of relapse (e.g., age, familiarity, lifestyle habits) [6]. Obesity is a chronic condition that requires continuous care, behavioral therapies and psychological support, so a multidimensional approach, appears to be a successful strategy for a weight loss program [10]. The main advantage of the therapeutic-rehabilitative intervention for obesity is the multidisciplinarity of the program in which the professionals involved work in synergy on the health status of the obese patient, who often has metabolic comorbidities and psychological disorders.

The current meta-analysis aims to compare the effects of the inpatient treatment phase (hospitalized weight loss programs) and during the outpatient phase on body weight and BMI 6 and 24 months of follow up treatment.

## Materials and methods

The present meta-analysis was conducted in accordance with the PRISMA (Preferred Reporting Items for Systematic Review and Meta-Analyses) statement [11].

It was performed through the following steps:

1. formulation of the review question: “inpatient and outpatient treatment for weight loss”;
2. definition of participants: obese patients;
3. search strategy for the identification of relevant intervention studies that included inpatient and outpatient treatment for weight loss;
4. analysis of the data through the meta-analysis;
5. data extraction was performed independently by three investigators (C.R, SP and C.G.) and discrepancies were resolved by a forth reviewer (M.R.).

### Search strategy and eligibility criteria

It has been carried out an electronic search using primarily Medline, Google Scholar, Embase, Central and Scopus and the Science Citation Index databases, without any language restriction. The search was carried out as follow: “weight loss inpatients” AND “residential program for obesity care” AND “obesity inpatient rehabilitation program” AND “treatment for obesity in hospital” AND “continuous care in the treatment of obesity” AND “residential weight loss program”. For each database, the investigators have considered the study published in the last 20 years.

Potentially eligible studies were: English written, that reported weight loss and BMI as primary outcomes, inpatients and outpatients obesity treatment at 6 and 24 months as follow up. All eligible studies included baseline and follow-up values, the mean change differences (∆-change) and relative standard deviation from baseline, and/or the mean difference among intervention groups vs. control group, concerning body weight and BMI and the sample size.

Studies in which diet were combined with pharmacological treatments or bariatric surgery were excluded.

### Analysis of the data and presentation of the outcomes

Intervention studies investigating the effectiveness at 6 and 12 months of inpatient and outpatient programs for weight loss in obese people were included. For each study, the following data were specified: first author and the year of publication, the country, the inclusion criteria, the sample, the dietary and parallel intervention, and the duration of the intervention. A meta-analysis for pooled estimate for aggregated data was performed.

### Risk of bias in individual studies

The risk of bias of each study was assessed using the Cochrane Collaboration Risk of Bias tool [12] and considering as factors contributing to the study quality the generation of the allocation sequence, the allocation concealment, the blinding of outcome data, the presence of incomplete data and the selective reporting. These factors were classified as low risk of bias, high risk of bias or unclear risk of bias. Studies with a low risk of bias for at least three items were held as good; studies with a low risk of bias for at least two items were considered as fair, and studies with a low risk for no item or only for one item were regarded as poor.

## Results

### Studies characteristics

**Fig. 1:**
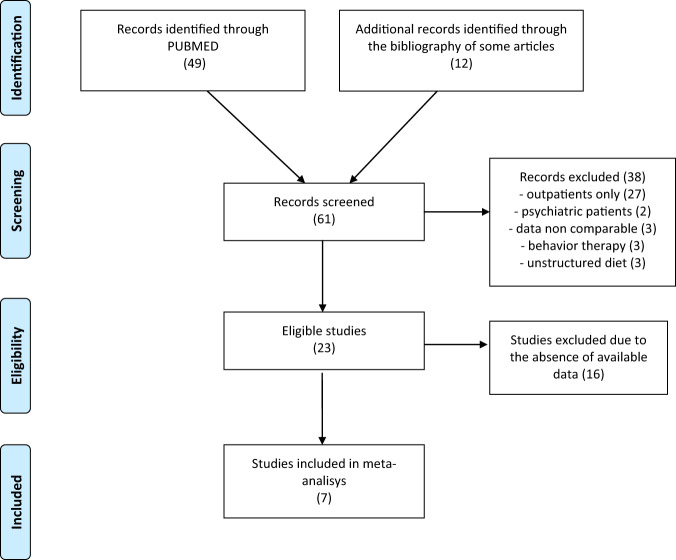
Flow diagram.

The literature search retrieved 61 articles through the database searching and, after applying the exclusion criteria a total of 23 remaining articles were fully analyzed. Of these 23, 7 studies were included in a meta-analysis. Figure 1 shows the study selection procedure.

The setting of the studies included in the analysis, refers to residential therapeutic-rehabilitative intervention, specifically for obese patients. Intervention period lasted from a minimum of 3 to a maximum of 280 weeks of follow up.

The seven studies included a total of 977 obese subjects both women and men. The intervention group and the control group are represented by the inpatient and outpatient dietary program, respectively. Mean of age was 38.5 ± 6.65 years and mean of body mass index (BMI) was 44 ± 2.40 kg/m^2^. The mean weight loss in the inpatients groups at 3 weeks was 4.38 ± 1.29 kg. The mean weight loss in the outpatient groups at 6 months was 11.29 ± 6.06 kg.

The data obtained from the studies included have been layered into two categories: short term (studies with follow-up of max 6 months) and long term (studies with follow-up up to 24 months).

### Studies characteristics

The literature search retrieved seven studies, as shown in Table 1. The table summarizes the studies that evaluated a weight loss intervention in inpatients and outpatients. In our analysis we considered obese patients with different profiles, such as different metabolic comorbidities and binge eating disorders (BED).

**Table 1:** Studies included in the meta-analysis.

First author, year	Country	Inclusion criteria	Sample	Dietary intervention	Non dietary intervention	Duration of the intervention
Maffiuletti, 2005	Italy	Age ≥ 18 years, BMI ≥ 35	64 (45 F, 19 M)	Energy restricted diet (1200–1800 kcal)	Nutritional education, psychological counseling, physical activity	3 weeks inpatient and follow up after 1 years (outpatient treatment).
Riva, 2006	Italy	Women aged 18–50 years, BMI > 40	ECT: 57 CBT: 54 NT: 52 Waiting list: 53	1200 Kcal (low calorie diet)	Cognitive-behavioral Therapy (CBT), Experiential cognitive therapy (CT)	6 weeks inpatient and follow up at 6 months (outpatient treatment)
Christiansen, 2006	Denmark	Individuals who had completed a minimum of 8 weeks of treatment at the weight loss camp	249 (180 F, 69 M)	2190 kcal/day	Physical activity 120 min /day, cognitive strategies	21 weeks (mean duration) + follow up to 2,3,4 years.
Martins, 2010	Norway	Age between 18 and 60 years old, BMI > 40 or >35 with comorbidities	A: 55 B: 30 C: 64 D: 57 (154 F, 52 M)	Bariatric surgery or low-calorie diet	Nutritional education, physical activity and psychotherapy.	B: 8–10 week in residential structure (RRC), 8 weeks at home, 4 weeks in RRC, 4–5 month at home, 2 weeks in RRS and 2 week every 6 months after the first year up to 5 years. C: 21 weeks in private health resort. Low calorie diet. D: Six months weight loss program at Hospital (hospital outpatient program)
Dalle Grave, 2013	Italy	Obese patients aged 18–65 years, BMI ≥ 40.0 kg or between 35 and 39.9 with comorbidity	88 (HPD 43, HCD 45). 51 F, 37 M	High protein diet (HPD) and high-carbohydrate diet (HCD), combined with cognitive behavior therapy (CBT).	Education with cognitive behavioral procedures, and strategies to enhance their adherence to lifestyle modification	3 weeks inpatients and 48 weeks outpatient treatment.
Cesa, 2013	Italy	Women aged 18–50 years, who met DSM-IV-TR criteria for BED for at least 6 months prior to the beginning of the study.	66 patients divided into three groups: ECT (virtual reality protocol): 27 CBT (cognitive behavior therapy): 20 IP (inpatient multimodal treatment): 19	Low calorie diet (1200 Kcal)	IP: Psychological support and physical training CBT: Psychological support and physical training. 15 additional cognitive behavior therapy. ECT: IP + CBT and 15 additional VR-enhanced cognitive behavior therapy	IP: 6 weeks and follow up at 1 year (outpatient treatment) CBT: 5 weeks and follow up at 1 year (outpatient treatment).ECT: 5 weeks and follow up at 1 year (outpatient treatment).
Calugi, 2017	Italy	Obese patients aged 18 to 65 years, BMI ≥ 40 or between 35 and 39.9 with comorbidity	88 (51 F, 37 M)	High Protein Diet (HPD) and high-carbohydrate diet (HCD) both 1200 kcal/day for women and 1500 kcal/day for men	Education with cognitive behavioral procedures, and strategies to enhance their adherence to lifestyle modification	The weight loss phase: 3 week of inpatients treatment Maintenance phase: 24 weeks (outpatient treatment)

*SBP* Systolic Blood Pressure (mmHg), *DBP* Diastolic Blood Pressure (mmHg), *Total C.* Total Cholesterol (mg/dl), *HDL C* High Density Lipoprotein Cholesterol (mg/dl), *LDL C* Low Density Lipoprotein Cholesterol (mg/dL), *FBG* Fast Blood Glucose (mg/dl), *WC* Waist circumference, *BMI* Body Mass Index, *WL* Weight Loss.

### Meta-analyzed data

#### Body weight

**Fig. 2:**
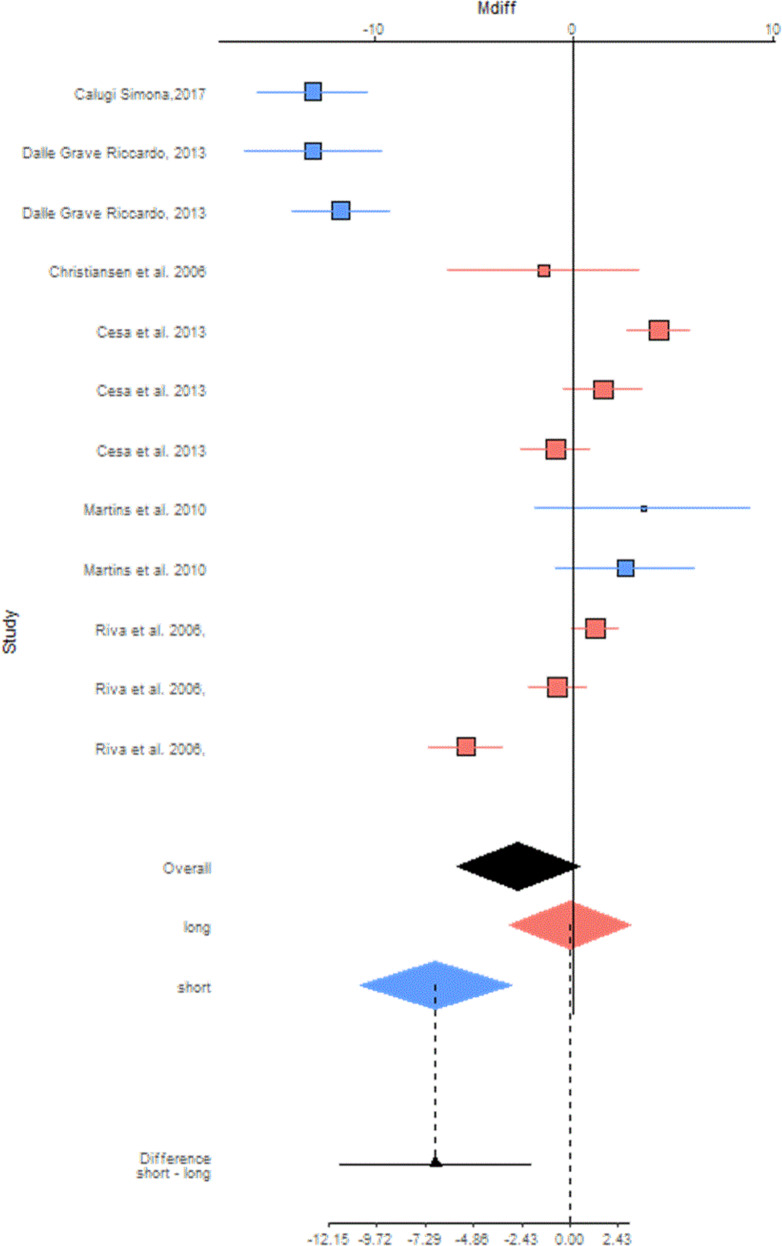
Forest plot studies included in body weight (kg) subgroup meta-analysis.

As shown by the blue diamond in Fig. 2, the meta-analyzed mean differences for random effect (MD) showed a statistically significant decrease in body weight of −6.94 kg for subjects undergo a short hospitalization compared to outpatients (95% CI: −10.71 to −3.17; *P* = 0.0003). The blue diamond refers to studies in which the treatment has a follow-up up to 6 months. Instead, as showed by the pink diamond, at 24 months the reduction of body weight was −0.19 kg (95% CI: −3.20 to 2.82; *P* = 0.9) for subjects who undergo a long hospitalization compared to an outpatient treatment (*p* = NS). The pink diamond considers studies in which the treatment has a follow-up from 6 to 24 months.

The black diamond showed Fig. 2 represents the overall effect of a hospitalization for weight loss without considering the time variable. The overall results showed that those hospitalized had a reduction of body weight of −2.82 kg compared to those who follow an outpatient course, but the decrease is not statistically significant (95% CI: −5.88 to +0.25; *P* = 0.07).

Moreover, the analysis highlighted the effects on weight loss the subjects undergo a short hospitalization had, compared to those who were hospitalized for a long time. The weight loss mean difference was −6.75 kg (95% CI: −11.58 to −1.93; *P* = 0.006).

#### Body mass index

**Fig. 3:**
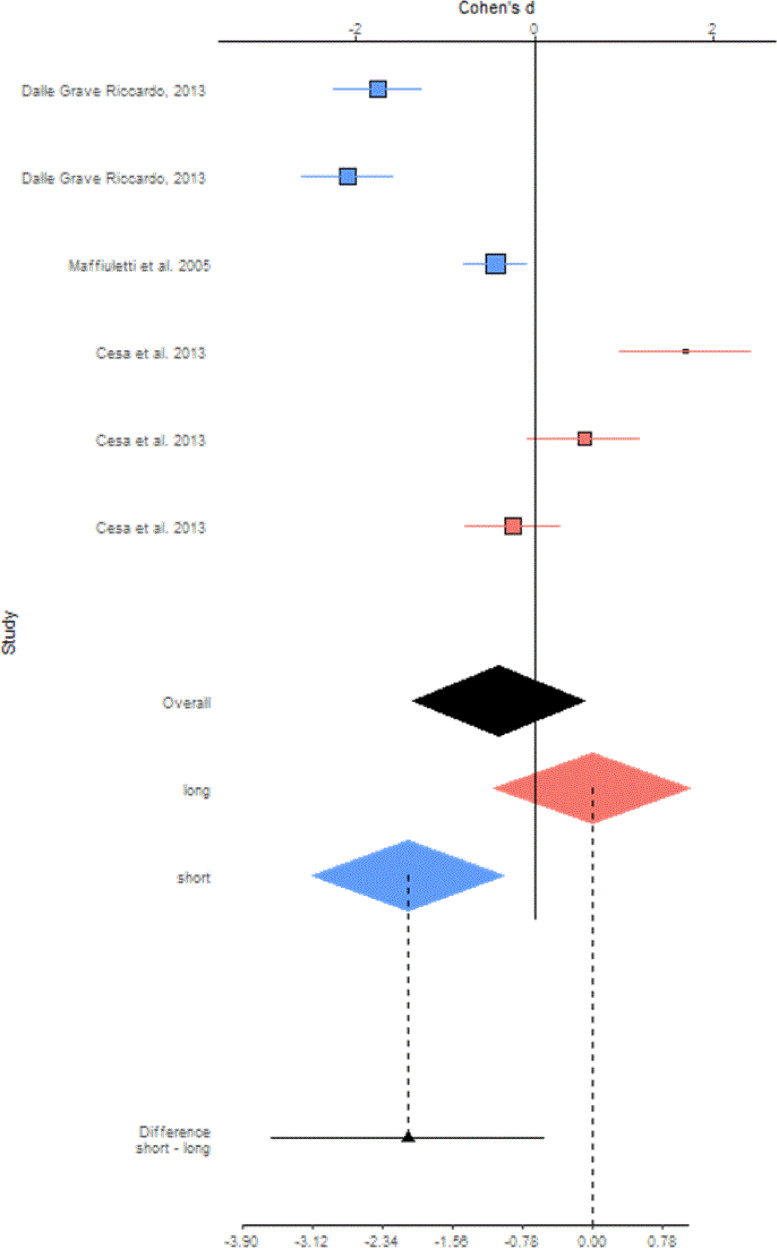
Forest plot studies included in body mass index (kg/m2) subgroup meta-analysis.

As previously reported, the blue diamond refers to studies in which the treatment has a follow-up up to 6 months, while the pink diamond considers studies with a duration from 6 to 24 months. As shown by the blue diamond in Fig. 3, the meta-analyzed mean differences for random effect (MD) showed a decrease in body mass index of −1.42 kg/m^2^ (95% CI: −2.48 to −0.35; *P* = 0.009) for subjects who undergo a short hospitalization compared to an outpatient treatment. On the contrary, as showed by the pink diamond, a long-term hospitalization did not reduce the body mass index significantly (0.64 kg/m^2^; 95% CI: −0.45 to 1.74; *P* = 0.25) for subjects who undergo a long hospitalization compared to outpatient.

The black diamond in Fig. 3 represents the overall effect of a hospitalization for weight loss without considering the time variable. The overall results showed that those who are hospitalized did not have a significant reduction of body mass index of −0.40 kg/m^2^ (95% CI: −1.37 to +0.57; *P* = 0.41), compared to those who follow an outpatient course. Finally, the analysis highlighted that subjects who undergo a short hospitalization had a reduction of BMI of −2.06 kg/m^2^ (95% CI: −3.59 to −0.53; *P* = 0.008). compared to those who were hospitalized for a long time.

## Discussion

This meta-analysis revealed that the subjects underwent a short hospitalization had greater benefit, compared to those who were hospitalized for a long time. A significant decrease on BMI and on body weight was recorded in subjects who undergo a short hospitalization compared to outpatients. In addition, this study did not report any reduction of body weight and BMI for subjects who undergo a long hospitalization compared to an outpatient.

Since all of the hospitalized studies are followed by outpatients treatment, what emerges is the fact that hospitalization at the beginning of obesity treatment is significantly better than only outpatients treatment.

These findings are an important matter of discussion, since in literature, studies about hospitalizations for the treatment of obesity are limited and the first dates back to the 1980s when the rate of obesity in the world began to rise [13].

One of the factors that could facilitate patients that undergo an inpatient weight loss treatment is the multidimensional approach based on physical activity and psychological support.

The importance of psychological therapy within hospital recovery for weight loss is analyzed for the first time in 2006 by Riva et al., who demonstrate how, during hospitalization, the use of cognitive behavioral therapy (CBT) or experiential cognitive therapy (ECT) are discriminating in the outpatient phase, especially in the group subjected to ECT, while nutritional therapy alone is less effective in the long term [14, 15]. The patients continue to lose in the follow-up phase, strengthening the evidence that ECT is a fundamental therapy in the hospitalization phase to maintain long-term results [14].

In any case, CBT or ECT are necessary to determine a constant weight loss in the outpatient phase [14–16]. Opposite, in the study by Maffiuletti et al., the patients, who carry out a 3-weeks hospitalization, register a minor weight loss and this is probably attributable to the fact that the authors do not apply either CBT or ECT but a simple psychological counseling that is not as effective [17].

If psychological support is also maintained in the outpatient phase, some patients may be able to maintain lifestyle changes not just for 1 year, but for another 5 years by decreasing cardiovascular risk factors [18].

Moreover, patients who received constantly under hospitalization different nutritional education sessions during the inpatient programs have demonstrated to continue to weight loss) [19].

In most of the studies included in the current review, the patients under hospitalization carry out physical activity every day; it is widely known that physical activity practice results in a weight loss greater than 20% in the context of a low-calorie diet compared to calorie restriction alone [20].

Although in the hospitalization phase, the patients practiced physical activity, in the outpatient phase, a lower weight loss compared to hospitalization were observed: in fact, 24 months after admission, the constancy of patients decreased, leading to a minimal weight loss in the outpatient phase and losing the benefits gained during the hospitalization in terms of cardiovascular risk reduction and other comorbidities [17, 19].

The decrease of body weight during hospitalization leads to a clear reduction in cardiovascular risk factors and an improvement in health conditions, regardless of the type of diet proposed. In fact, even if the LCD < 1200 kcal leads to a greater weight loss than a normocaloric diet, the latter one is able to generate an important weight loss too, potentially reproducible in the outpatient phase. For both types of diet, however, the initial hospitalization phase is essential for the patient’s re-education to change his lifestyle. This is evident from the comparison of the studies conducted by Martins [21] and Christiansen [19] in which patients undergo 21 weeks of hospitalization but with two different kind of diets (LCD and over 2000 kcal, respectively): Martins patients lose weight faster, but in both cases the loss is constant and maintained in the follow up. In addition, Martins further strengthens the thesis that hospitalization is essential for losing weight in the outpatient phase: in his study, in fact, he inserts a group that does not carry out hospitalization but only outpatient treatment: the weight loss of this group is negligible, compared to those who have previously been hospitalized [21].

In addition, the recovery is essential for the patient’s lifestyle re-education. This is evident in the study conducted by Martins in which patients who underwent a previous hospitalization with personalized diet, psychological support and physical activity, in the outpatient phase gained a greater weight loss than those who start the outpatient diet without having experienced the hospitalization phase [21]. Therefore, it’s evident that the hospitalization phase is essential to ensure that the patient has a better chance of continuing to lose weight in the outpatient phase.

Together with nutritional therapy, physical activity and psychological support are fundamental. In fact, once out of the hospitalization the patient is exposed daily to an obesogenic environment: the use of cars, lifts and escalators do not favor movement and the increased hours of sedentary work also do not improve the state of health [22]; epidemiological studies on working conditions have shown an association between sedentary work and a higher BMI [23]. In addition, the continuous proposal of palatable but ultra-processed, caloric, full of sugar, salt and fat foods greatly increase the energy intake, leading to a rapid weight gain [24].

For this reason, the objectives of the hospitalization should be multidimensional: nutritional, of physical activity and psychological. The obese patient should set himself realistic weight goals to avoid dissatisfaction and abandonment of diet therapy [25]. The type of diet applied appears, from the analyzed data, to be less relevant than the setting of CBT or ECT, which are fundamental for the constant maintenance of weight loss even in follow up phase. The results obtained, also shows a lower average of weight loss in longer hospitalization and this is probably due to the fact that in the first weeks the weight loss is faster. In addition, it is possible that in the 21-week studies in which patients practiced physical activity, body composition changed in favor of lean mass, but this data is not available.

The maintenance of weight loss is not only not related to the type of diet therapy set, but, according to Björvel, it is more likely to occur if a first phase of hospitalization is carried out and if, after this first phase, continuous treatment is carried out in the outpatient phase [26]. The hospitalization is useful for decreasing body weight and cardiovascular risk factors. In the outpatient phase, for a continuous and durable weight loss, psychological support and constant contact with the patient is important to maintain results in the long term [16].

The present meta-analysis has several limitations. Firstly, the studies considered have different follow-up lengths and are not all randomized clinical trial studies. The heterogenicity in the design of the study represent a critical issue, but also a great start point, since this meta-analysis represent the sum of inpatient and outpatient phase for a comparison.

## Conclusions

The results of this meta-analysis showed that an impatient treatment at the beginning of any obesity program is significantly better than only outpatients treatment.

Specifically, the subjects underwent a short hospitalization had greater benefit, compared to those who were hospitalized for a long time.
